# The Association of Four-Limb Blood Pressure with History of Stroke in Chinese Adults: A Cross-Sectional Study

**DOI:** 10.1371/journal.pone.0139925

**Published:** 2015-10-09

**Authors:** Hong Guo, Fengyu Sun, Lihang Dong, Huiying Chang, Xingbo Gu, Haiyu Zhang, Lijiang Sheng, Ye Tian

**Affiliations:** 1 Department of Cardiology, The First Affiliated Hospital, Cardiovascular Institute, Harbin Medical University, Harbin 150001, P.R. China; 2 Department of Pathophysiology, Key Laboratory of Cardiovascular Pathophysiology, Harbin Medical University, Harbin 150081, P.R. China; 3 Key Laboratory of Cardiovascular Medicine Research, Ministry of Education, Harbin Medical University, Harbin 150081, P.R. China; 4 Heilongjiang Academy of Medical Sciences, Harbin Medical University, Harbin 150081, P.R. China; Shanghai Institute of Hypertension, CHINA

## Abstract

**Objective:**

We investigated the association of ankle-brachial blood pressure index (ABI), interarm blood pressure (BP) difference and interankle BP difference, obtained by simultaneous four-limb BP measurement, with history of stroke in a Chinese adult population.

**Methods:**

This cross-sectional study included 1485 participants aged ≥35 years in the framework of the China Hypertension Survey. We performed simultaneous four-limb BP measurement using oscillometric devices with the participants in the supine position and calculated ABI and interarm/interankle BP differences between the 4 limbs. Logistic regression analysis was used to estimate the association of these BP parameters and other factors with a history of stroke.

**Results:**

In univariate analyses, participants with ABI <0.9, interarm BP difference ≥15 mmHg, and interankle BP difference ≥10 mmHg had a higher prevalence of stroke than those without (*p* < 0.0001, *p* = 0.0152, *p* = 0.002, respectively). Multiple logistic regression analyses suggested, ABI <0.9 was independently associated with a history of stroke after adjustment for interarm BP difference ≥15 mmHg, interankle BP difference ≥10 mmHg, and traditional risk factors for stroke (*p* = 0.001). An interankle BP difference ≥10 mmHg was associated with prior stroke after the two variables of hypertension and ABI were removed from the logistic regression model (*p* = 0.0142). Net reclassification improvement analysis showed that inclusion of interankle BP difference ≥10 mmHg to the independent risk factors (age, family history of stroke, hypertension, and ABI) improved net reclassification by 11.92%.

**Conclusion:**

ABI <0.9 is an independent risk factor for stroke prevalence in Chinese adults and it just detected a small propotion of paticipants. The addition of interankle BP difference ≥10 mmHg to the independent risk factors for stroke may improve the prediction of stroke.

## Introduction

Stroke is the leading cause of death and long-term disability in Chinese adults [1]. The total stroke incidence rate has declined over the past two decades in the USA, but in contrast has increased annually by 6.5% in China [2, 3]. High blood pressure (BP) is the primary risk factor for stroke and contributes to approximately two-thirds of the cerebrovascular disease burden [3–5]. When peripheral artery disease (PAD) is present in arteries in the upper extremities, the reduced BP in the diseased arm may not only possibly delay the diagnosis of hypertension, but also lead to delays in the implementation of interventions to decrease the risk of stroke.

In recent years, it has been proposed that four-limb BP measurements may be useful for cardiovascular prediction and prevention [6]. Current technology allows simultaneous BP measurement in all four limbs [7, 8], providing a comprehensive evaluation of BP and generating accurate measurements of BP ratios and differences between the four limbs, such as the ankle-brachial BP index (ABI) and the interarm and interankle BP differences. However, the epidemiology of BP ratios and differences obtained from simultaneous BP measurements has not been well characterized, and the relationship between abnormal four-limb BP parameters determined from simultaneous BP measurements and stroke has received little attention. It is important to evaluate the relevance of these BP-derived indexes to stroke, as the identification of individuals with abnormal BP is both simple and quick, and may afford the opportunity to reduce aggressively the risk of stroke with preventive therapy.

The ABI is a well-documented diagnostic tool for PAD in the lower extremities [9], but also provides information beyond PAD. The results of the population-based Heinz-Nixdorf study in Germany filled a gap in the literature by confirming that ABI is an independent risk marker for stroke [10]. However, we have little knowledge about the association between ABI and stroke in the Chinese population.

Uncertainty remains about the association between the interarm BP difference and stroke because of limited and inconsistent data. A recent systematic review found that a systolic interarm difference of 15 mm Hg or more, based on a non-simultaneous measurement technique, is also associated with the presence of cerebrovascular disease [11]. However, no association was noted when the analysis was restricted to studies of tens or hundreds of participants using a simultaneous measurement method. A recent meta-analysis has suggested that future epidemiologic studies of between-arm BP differences should use a repeated simultaneous measurement method [12]. Given this, the present study utilized a simultaneous measurement method in a larger sample to investigate the association between an interarm BP difference of 15 mm Hg or more and stroke.

Although the interankle BP difference can predict the overall risk for cardio-cerebrovascular events in general [6, 13], few studies have demonstrated an association between the interankle BP difference and stroke. It is important to clarify whether an elevated interankle BP difference would identify some low-risk individuals with an increased likelihood of stroke.

In the present cross-sectional study we investigated the odds of prior stroke based on conventional risk factors for stroke and simultaneous four-limb BP ratios and differences, in order to assess the possible utility of these BP parameters in predicting stroke risk in Chinese adults.

## Materials and Methods

### Study Population

This was a cross-sectional study conducted in two Shuangcheng Manchu townships, within the framework of the China Hypertension Survey supported by the Chinese Ministry of Science and Technology [14]. Shuangcheng is located in Heilongjiang Province, in northeast China. From December 2014 to November 2014, an epidemiologic study was conducted there to evaluate cardiovascular disease and risk among adults aged ≥35 years, selected using a simple random sampling method [14]. The Ethics Committee of the First Affiliated Hospital of Harbin Medical University approved the study protocol. All subjects gave written informed consent.

A total of 1591 subjects were enrolled (participation rate 80%). We excluded 106 subjects because four-limb BP measurement was not performed (n = 34), no blood test was performed (n = 17), or miscellaneous information was missing (n = 55). Thus, the final number of participants included in the present analysis was 1485.

### Study protocol and evaluation criteria

Each participant underwent a physical examination and was administered a standardized questionnaire by a trained interviewer. The questionnaire included data on age, sex, smoking status (past or present smoker, number of cigarettes smoked daily, and smoking years), alcohol intake (past or present alcohol drinker, amount and times of alcohol drunk daily, and drinking years), history of stroke, hypertension and diabetes, and family history of stroke. In this study, smoking was defined as smoking every day for more than 1 year. Alcohol intake was defined as having at least one drink every day.

An experienced physician measured each participant’s BP three times consecutively using a validated Omron HBP-1300 oscillometric BP monitor (Omron, Kyoto, Japan) on the right arm, after the subject had rested for ≥5 minutes in a sitting position. Hypertension was defined as a sitting systolic BP (average of 3 readings) of ≥140 mmHg or diastolic BP ≥90 mmHg or the use of antihypertensive drugs.

Four-limb BP was measured using two Watch BP Office ABI devices (Microlife, Widnau, Switzerland), which use an oscillometric technique that has been validated previously [15]. Trained technicians and physicians placed the pressure cuffs on both arms and both ankles, and performed the measurements after the subject had rested for approximately 10 minutes in the supine position. The device simultaneously and automatically measured the BPs 3 times. ABI was reported for each side, and the lower average reading of ABI was used for analysis. ABI <0.9 was considered as abnormal. We calculated the interarm and interankle BP differences as the average and absolute systolic values of the difference between the right and left brachial BPs and between the right and left ankle BPs, respectively. An elevated interarm difference was defined as ≥15 mmHg and an elevated interankle difference as ≥10 mmHg.

Body mass index (BMI) was calculated as weight in kilograms divided by height in meters squared. Normal BMI was defined as <24 kg/m^2^.

Venous blood samples were drawn after overnight fasting for the measurement of plasma glucose, serum total cholesterol (TC), low-density lipoprotein cholesterol (LDL-C), high-density lipoprotein cholesterol (HDL-C), and triglyceride (TG) levels. Impaired fasting plasma glucose and dyslipidemia were defined according to the guidelines [16, 17].

Stroke was diagnosed by a neurologist in a hospital at or above the county level based on the self-reported history of stroke and cranial computed tomography or magnetic resonance imaging. Nonfatal ischemic stroke, hemorrhagic stroke, and TIA were all included. Of the study participants diagnosed with stroke, 6 (4.4%) had hemorrhagic stroke (including 2 with subarachnoid hemorrhage),107 (78.7%) had ischemic stroke, and 23 (16.9%) had TIA.

Diabetes mellitus was defined as a fasting plasma glucose concentration ≥7.0 mmol/L, prior diagnosed diabetes mellitus, or the use of antidiabetic agents [16].

### Statistics

Continuous variables are expressed as mean ± standard deviation (SD), except for fasting plasma glucose, interarm BP difference, and interankle BP difference, which are expressed as median (interquartile range [IQR]) because of the high skewness. Categorical variables are presented as proportions. Means and proportions were compared using the Student *t*-test and chi-square test, respectively. Non-normally distributed variables were compared by non-parametric. Logistic regression was performed to assess the association of ABI, interarm BP difference and interankle BP difference with the prevalence of stroke. Adjustment was made for age, sex, ethnicity, family history of stroke, BMI, smoking status, alcohol use, hypertension, TC, LDL-C, HDL, TG and fasting plasma glucose levels as potential confounders. The incremental value of the interankle BP difference was tested by comparing the area under the receiver operator characteristic curve (ROC_AUC_). ROC_AUC_ was estimated for a regression model with age, family history of stroke, hypertension and ABI as a categorized variable, and for a model with age, family history of stroke, hypertension, ABI and interankle BP difference as a categorized variable, both in relation to a dichotomous outcome of history of stroke (with versus without). Net reclassification improvement (NRI) was computed to indicate the proportion of subjects reclassified correctly (NRI >0) or incorrectly (NRI <0) into various risk categories. NRI was calculated for participants when adding categorized interankle BP difference (<10 mmHg versus ≥10 mmHg) to the age, family history of stroke, hypertension and ABI model for predicting stroke prevalence. The new model was formed by adding interankle BP difference as a categorized variable to stroke risk categories (<10%, 10–20%, 20–30%, >30%) based on risks predicted using the model based on age, family history of stroke, hypertension and ABI, where interankle BP difference ≥10 mmHg led to upward or downward risk category shift, respectively. The NRI was calculated by summing up the percentage being net correctly reclassified [18, 19]. Whether NRI was significantly larger than zero was analyzed using the Z-test [18, 19]. SAS software (version 9.13, SAS Institute, Cary, NC) and the R language (R Development Core Team) were used for database management and statistical analysis. A 2-sided *p*-value <0.05 was considered to be significant.

## Results

The 1485 participants (740 men, 49.8%) were aged 54.76 ± 11.50 years, and included 136 (9.16%) participants with stroke, 621 (41.81%) with hypertension, and 111 (7.47%) with diabetes mellitus. Manchu ethnicity accounted for 50.23% of the population (Table 1). The mean ABI was 1.17 ± 0.11 in the study population. Males had higher ABI than females (1.19 ± 0.1 vs. 1.16 ± 0.11, *p* < 0.0001). The prevalence of ABI < 0.9 was 1.75%, with no difference between females (2.01%) and males (1.49%). The number of cases with ABI <0.9 in the left and right limbs was 12 and 14, respectively. The median interarm BP difference was 3.3 (IQR 1.3, 5.7) mmHg; 33 (2.22%) participants had an interarm BP difference ≥15 mmHg, and 119 (8.01%) had an interarm BP difference ≥10 mmHg. The median interankle BP difference was 5.7 (IQR 2.3, 11.7) mmHg; 254 (17.10%) participants had an interankle BP difference ≥15 mmHg, and 460 (30.98%) had an interankle BP difference ≥10 mmHg. The prevalence of interarm and interankle BP differences ≥15 mmHg was similar between the sexes (*p* = 0.8757, *p* = 0.8027, respectively) (Table 1).

**Table 1:** Sex-stratified baseline characteristics of the study population.

Baseline variable	All (*n* = 1485)	Women (*n* = 745)	Men (*n* = 740)	*p*
**Age (years), mean ± SD**	**54.76 ± 11.50**	**53.33 ± 11.19**	**56.2 ± 11.63**	**<0.0001**
**Manchu ethnicity, *n* (%)**	**746 (50.23)**	**382 (51.28)**	**364 (49.19)**	**0.4215**
**BMI (kg/m** ^**2**^ **), mean ± SD**	**24.69 ± 3.79**	**24.90 ± 3.91**	**24.48 ± 3.65**	**0.0316**
**Use of antihypertensive drugs, *n* (%)**	**265 (17.85)**	**147 (19.73)**	**118 (15.95)**	**0.0567**
**Stroke, *n* (%)**	**136 (9.16)**	**58 (7.79)**	**78 (10.54)**	**0.0657**
**Hypertension, *n* (%)**	**621 (41.81)**	**297 (39.87)**	**324 (43.78)**	**0.1259**
**Family history of stroke, *n* (%)**	**333 (22.42)**	**178 (23.89)**	**155 (20.95)**	**0.1734**
**Diabetes mellitus, *n* (%)**	**111 (7.47)**	**50 (6.71)**	**61 (8.24)**	**0.2617**
**Systolic BP on higher arm side(mmHg), mean ± SD**	**133.13± 20.41**	**130.89± 20.40**	**135.38±20.18**	**<0.0001**
**Diastolic BP on higher arm side(mmHg), mean ± SD**	**81.17 ± 10.45**	**79.15 ± 9.77**	**83.19 ± 10.72**	**<0.0001**
**Interarm BP (mmHg):**				
** Systolic BP, median (IQR)**	**3.3 (1.3–5.7)**	**3.3 (1.3–5.7)**	**3.0 (1.3–5.7)**	**0.7852**
** Systolic ΔBP ≥15 mmHg, *n* (%)**	**33 (2.22)**	**17 (2.28)**	**16 (2.16)**	**0.8757**
** Systolic ΔBP ≥10 mmHg, *n* (%)**	**119 (8.01)**	**67 (8.99)**	**52 (7.03)**	**0.1629**
**Interankle BP (mmHg):**				
** Systolic BP, median (IQR)**	**5.7 (2.3–12)**	**5.7 (3.7–12.3)**	**5.7(2.3–11.7)**	**0.6598**
** Systolic ΔBP ≥15 mmHg, *n* (%)**	**254 (17.10)**	**133 (17.85)**	**121 (16.35)**	**0.4425**
** Systolic ΔBP ≥10 mmHg, *n* (%)**	**460 (30.98)**	**233 (31.28)**	**227 (30.68)**	**0.8027**
**ABI, mean ± SD**	**1.17 ± 0.11**	**1.16 ± 0.11**	**1.19 ± 0.1**	**<0.0001**
**ABI <0.9, *n* (%)**	**26 (1.75)**	**15 (2.01)**	**11 (1.49)**	**0.4389**

Values are presented as the mean ± standard deviation, median (interquartile range) or *n* (%). Abbreviations: ABI, ankle–brachial index; BMI, body mass index; BP, blood pressure; Δ, difference; IQR, interquartile range; SD, standard deviation.

The ABI in participants with a history of stroke was significantly lower than that in participants without a history of stroke (*p* = 0.0004). The interankle BP difference in participants with a history of stroke was significantly higher than that in those without (*p* = 0.0024). The interarm BP difference did not differ significantly between the two groups. The prevalence of ABI <0.9, interarm BP difference ≥15 mmHg, and interankle BP difference ≥10 mmHg was significantly higher in participants with a history of stroke than in those without (*p* < 0.0001, *p* = 0.0152, *p* = 0.0020, respectively) (Table 2).

**Table 2:** Comparison of the characteristics of the participants with and without a history of stroke.

Variable	Without history of stroke (*n* = 1349)	With history of stroke (*n* = 136)	*p*
**Age (years), mean ± SD**	**53.76 ± 11.26**	**64.4 ± 9.14**	**<0.0001**
**Manchu ethnicity, *n* (%)**	**672 (49.74)**	**76 (55.15)**	**0.2294**
**BMI (kg/m** ^**2**^ **), mean ± SD**	**24.71 ± 3.69**	**24.49 ± 4.61**	**0.5995**
**Family history of stroke, *n* (%)**	**296 (21.94)**	**37 (27.21)**	**0.8934**
**Hypertension, *n* (%)**	**524 (38.71)**	**97 (71.32)**	**<0.0001**
**Use of antihypertensive drugs, *n* (%)**	**204 (15.12)**	**61 (44.85)**	**<0.0001**
**Diabetes mellitus, *n* (%)**	**97 (7.19)**	**14 (10.29)**	**0.1896**
**Systolic BP on higher arm side (mmHg), mean ± SD**	**131.68 ± 19.52**	**147.5 ± 23.31**	**<0.0001**
**Diastolic BP on higher arm side (mmHg), mean ± SD**	**80.7 ± 10.24**	**85.8 ± 11.41**	**<0.0001**
**Interarm BP (mmHg):**			
** Systolic BP, median (IQR)**	**3.0 (1.3–5.7)**	**3.7 (1.6–5.7)**	**0.2433**
** Systolic ΔBP ≥15 mmHg, *n* (%)**	**26 (1.93)**	**7 (5.15)**	**0.0152**
** Systolic ΔBP ≥10 mmHg, *n* (%)**	**104 (7.71)**	**15 (11.03)**	**0.1741**
**Interankle BP (mmHg):**			
** Systolic BP, median (IQR)**	**5.3 (2.3–11.3)**	**8.7 (3.3–14.0)**	**0.0024**
** Systolic ΔBP ≥15 mmHg, *n* (%)**	**225 (16.68)**	**29 (21.32)**	**0.1704**
** Systolic ΔBP ≥10 mmHg, *n* (%)**	**402 (29.80%)**	**58 (42.65)**	**0.0020**
**ABI, mean ± SD**	**1.18 ± 0.1**	**1.13 ± 0.13**	**0.0004**
**ABI <0.9, *n* (%)**	**17 (1.26)**	**9 (6.62)**	**<0.0001**
**FBG (mmol/L), median (IQR)**	**5.2 (4.9–5.6)**	**5.3 (5.0–5.9)**	**0.0040**
**TC (mmol/L), mean ± SD**	**4.89 ± 0.96**	**5.19 ± 1.04**	**0.0012**
**HDL-C (mmol/L), mean ± SD**	**1.48 ± 0.35**	**1.41 ± 0.32**	**0.0127**
**LDL-C (mmol/L), mean ± SD**	**2.83 ± 0.81**	**3.14 ± 0.81**	**<0.0001**
**TG (mmol/L), mean ± SD**	**1.29 ± 0.87**	**1.43 ± 0.82**	**0.0649**

Values are presented as the mean ± standard deviation, median (interquartile range) or *n* (%). Abbreviations: ABI, ankle–brachial index; BMI, body mass index; BP, blood pressure; Δ, difference; FBG, fasting blood glucose; HDL-C, high-density lipoprotein cholesterol; IQR, interquartile range; LDL-C, low-density lipoprotein cholesterol; SD, standard deviation; TC, total cholesterol; TG, triglycerides.

**Fig 1:**
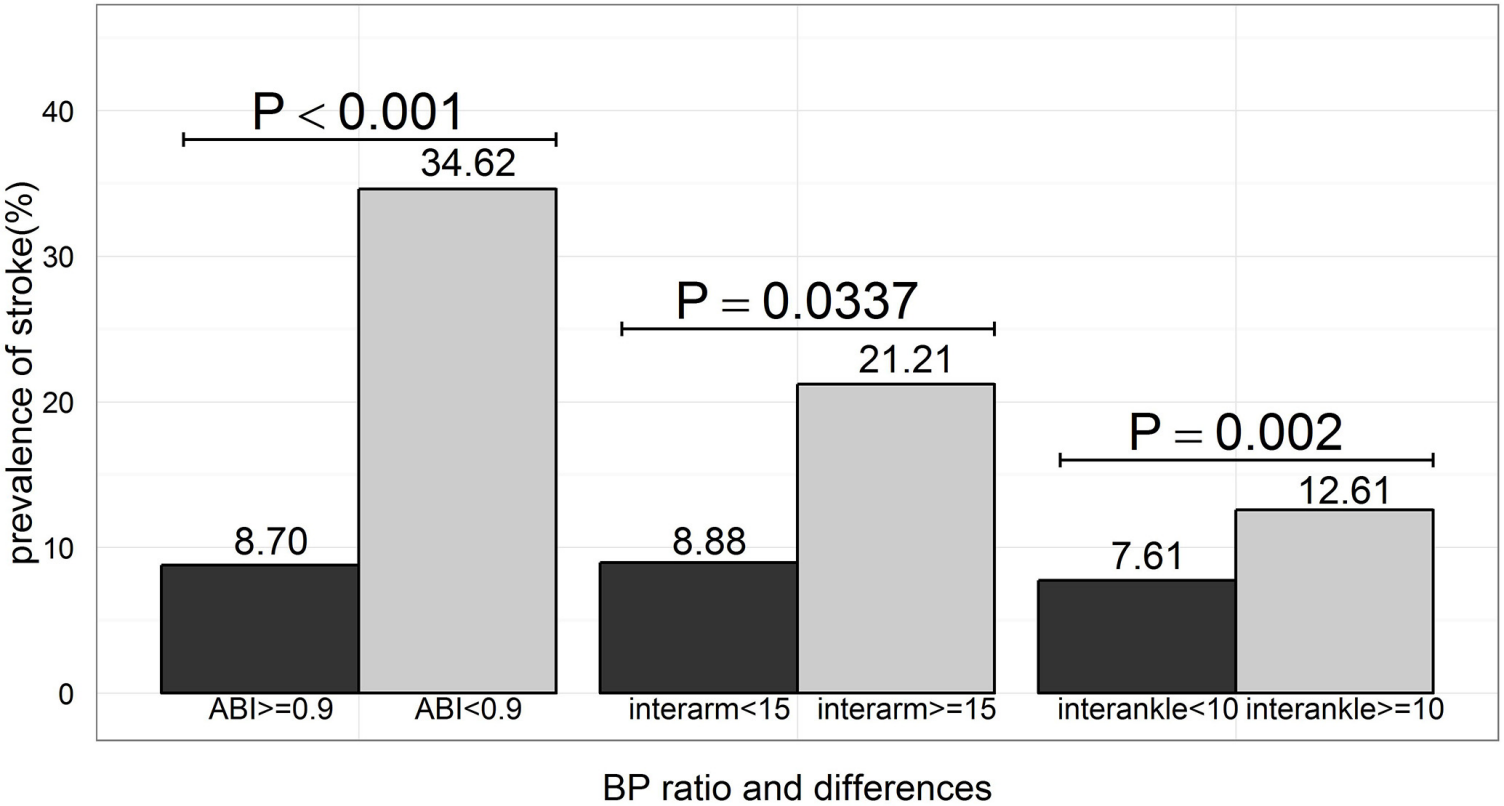
Prevalence of stroke stratified by the ankle-brachial blood pressure index (ABI) and interarm and interankle blood pressure (BP) differences. The prevalence of stroke was stratified according to the ABI (≥0.9 vs. <0.9, left) interarm BP difference (<15 mmHg vs. ≥15 mmHg, middle), and interankle BP difference (<10 mmHg vs. ≥10 mmHg, right). The *p*-value (Chi-square test) is given for each comparison. Numbers above the bars are rates of prior stroke.

Fig 1 shows the prevalence of stroke stratified according to the ABI and interarm and interankle BP differences. Participants with an interarm BP difference ≥15 mmHg had a higher prevalence of stroke than those with an interarm BP difference <15 mmHg (21.21% vs. 8.88%, *p* = 0.0337). Participants with an interankle BP difference ≥10 mmHg had a higher prevalence of stroke than those with an interankle BP difference <10 mmHg (12.61% vs. 7.61%, *p* = 0.002). Participants with an ABI <0.9 had a higher prevalence of stroke than those with an ABI ≥0.9 (34.62% vs. 8.70%, *p* < 0.0001).

**Fig 2:**
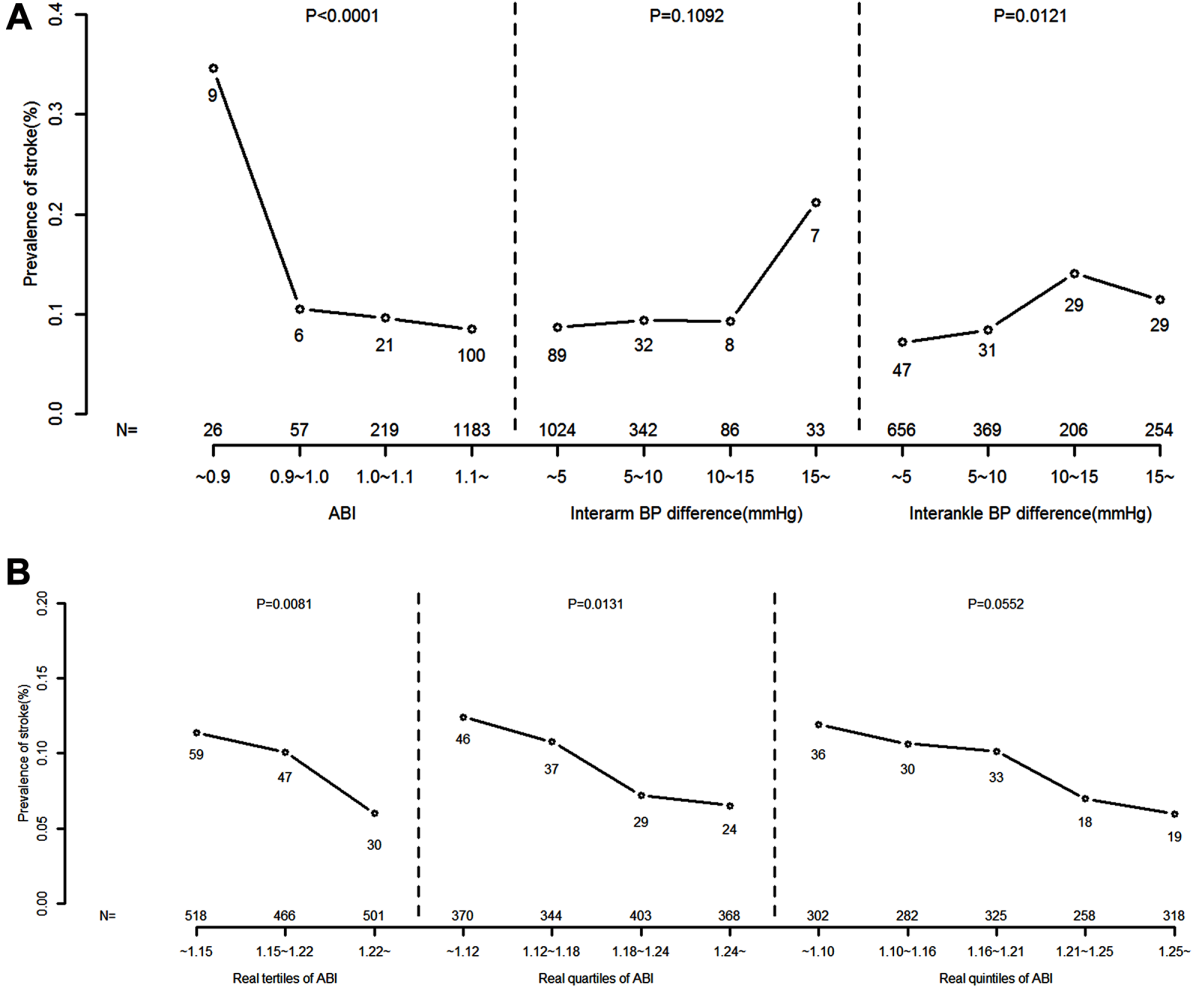
Prevalence of stroke stratified by the ankle-brachial blood pressure index (ABI) and interarm and interankle blood pressure (BP) differences. The numbers of strokes and participants are given alongside the symbols and at the bottom, respectively. *P*-values are given for trends in each group. A: The prevalence of stroke for different cutoff points of the ABI, interarm BP difference and interankle BP difference. Open circles represent the prevalence of stroke for ABI (left), interarm BP difference (middle) and interankle BP difference (right). B: The prevalence of stroke stratified according to real tertiles/quartiles/quintiles of the ABI distribution. Open circles represent the prevalence of stroke for real tertiles (left), quartiles (middle) and quintiles of the ABI distribution (right).

Categorical analyzes showed a curvilinear relationship between the prevalence of stroke and each of the ABI, interarm BP difference and interankle BP difference (Fig 2A). The prevalence of stroke stratified according to the real tertiles/quartiles/quintiles of the ABI distribution is shown in Fig 2B. There was a general trend for the prevalence of stroke to increase with decreasing tertiles and quartiles of the ABI (*p* = 0.0081, *p* = 0.0131, respectively).

**Fig 3:**
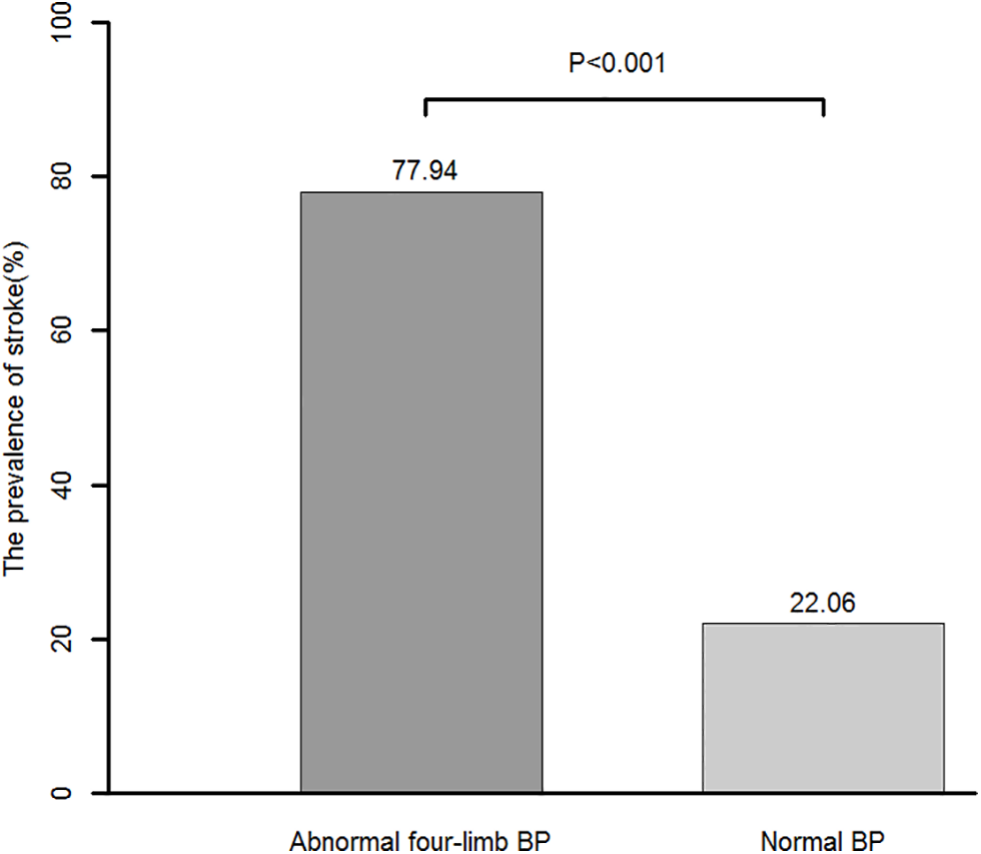
Abnormal four-limb blood pressure (BP) measurements and prevalence of stroke. Numbers above the bars are the rates of prior stroke. The *p*-value for comparison between the two groups was determined using the chi-square test.

Among the participants with a history of stroke, only 22.06% had no abnormalities in the four limb BP parameters; the other 77.94% had at least one of an interarm systolic BP difference ≥15 mmHg, interankle systolic BP difference ≥10 mmHg, ABI <0.9, systolic BP on the higher arm side ≥140 mmHg, or diastolic BP on the upper arm side ≥90 mmHg (Fig 3).

**Fig 4:**
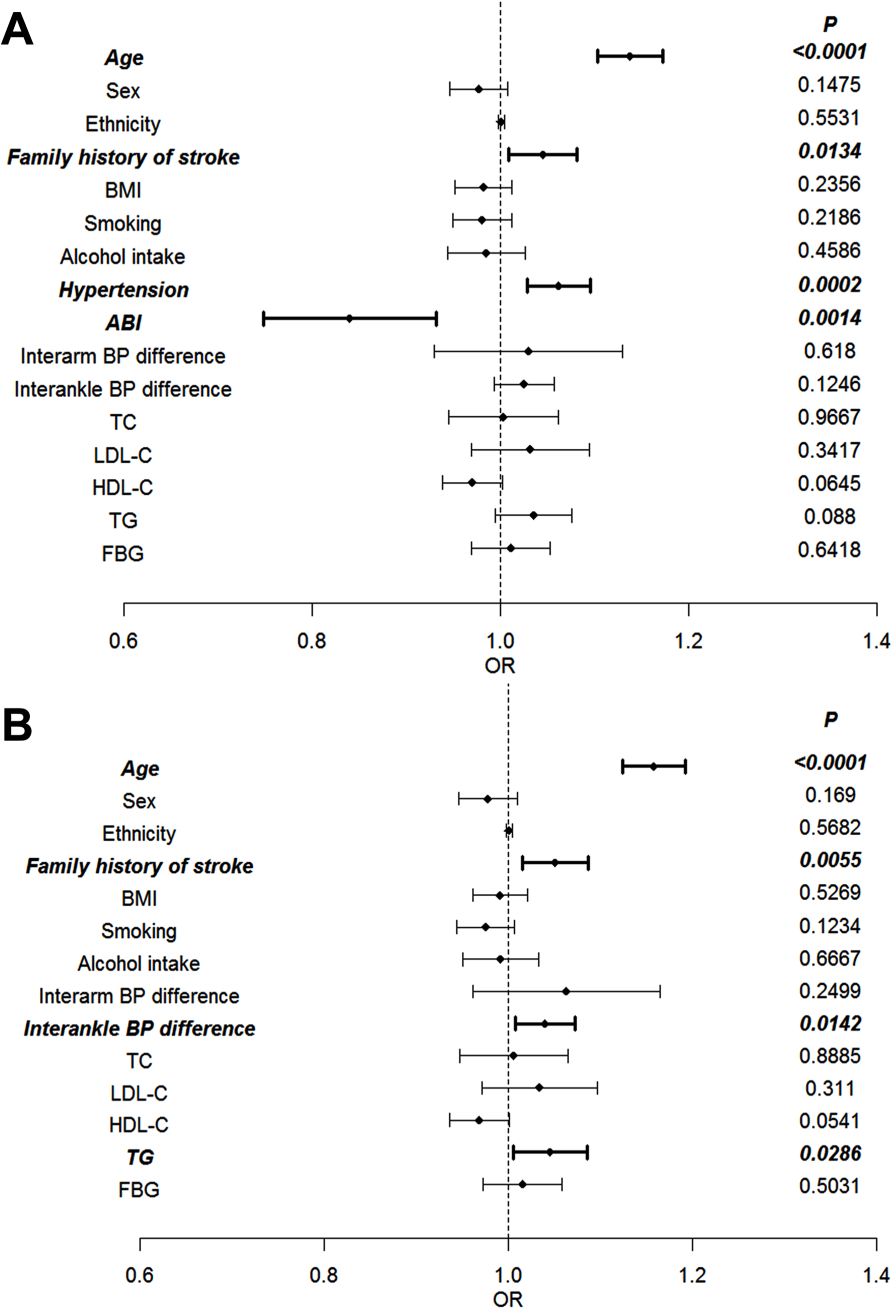
Multivariate logistic regression analysis of factors associated with the prevalence of stroke. The variables included in the analysis were sex (0, female; 1, male), age (0, 35–55 years; 1, over 55 years), ethnicity (0, Han; 1, Manchu), smoking (1, smoking every day for more than one year; 0, otherwise), alcohol intake (1, once at least every day; 0, otherwise), hypertension (1, systolic blood pressure ≥140 mmHg or diastolic blood pressure ≥90 mmHg, or the use of antihypertensive drugs; 0, otherwise), body mass index (0, <24 kg/m^2^; 1, ≥24 kg/m^2^), ankle-brachial blood pressure index (0, <0.9; 1 ≥0.9), interarm blood pressure difference (0, <15 mmHg; 1, ≥15 mmHg), interankle blood pressure difference (0, <10 mmHg; 1, ≥10 mmHg), total cholesterol (1, ≥5.21 mmol/L; 0, otherwise), triglycerides (1, ≥1.7 mmol/L; 0, otherwise), low-density lipoprotein cholesterol (1, ≥3.3 mmol/L; 0, otherwise), high-density lipoprotein cholesterol (0, <0.9 mmol/L; 1, otherwise), fasting blood glucose (1, ≥6.0 mmol/L; 0, otherwise). A: Adjusted co-variables with *p* < 0.05 in the univariate analysis and other well-documented risk factors for stroke (smoking status, alcohol intake and family history of stroke) were introduced into the multiple logistic regression models. B: Adjusted covariables with *p* < 0.05 in the univariate analysis, but excluding hypertension and ankle-brachial blood pressure index, and other well-documented risk factors for stroke (smoking status, alcohol intake and family history of stroke) were introduced into the multiple logistic regression models. Abbreviations: ABI, ankle-brachial blood pressure index; BMI, body mass index; BP, blood pressure; FBG, fasting blood glucose. HDL-C, high-density lipoprotein cholesterol; LDL-C, low-density lipoprotein cholesterol; TC, total cholesterol; TG, triglycerides.

After adjusting for ABI, interarm BP difference ≥15 mmHg, interankle BP difference ≥10 mmHg, and other classic confounding risk factors for stroke (age, sex, ethnicity, family history of stroke, BMI, smoking, alcohol, hypertension, TC, LDL-C, HDL, TG and fasting plasma glucose levels), multiple logistic regression analysis showed that ABI <0.9 was independently associated with a history of stroke (*p* = 0.001). In addition, age, hypertension and family history of stroke were also independently associated with stroke (*p* < 0.001, *p* < 0.001, *p* = 0.013, respectively) (Fig 4A). Interarm BP difference ≥15 mmHg and interankle BP difference ≥10 mmHg were not independently associated with a history of stroke (*p* = 0.618, *p* = 0.125, respectively). However, after the two variables of hypertension and ABI were removed from the multiple logistic regression analysis, interankle BP difference ≥10 mmHg was independently associated with stroke (*p* = 0.0142) (Fig 4B).

Although the results of the multivariate logistic regression analysis indicated that ABI was inversely related to stroke prevalence and that ABI <0.9 is an independent risk factor for stroke in the general Chinese population, it was noticeable that the prevalence of ABI <0.9 in the study population (only 1.75%) was much lower than that of a history of stroke (9.16%). This would suggest that ABI <0.9 may not be a sensitive predictor of stroke, in agreement with the findings of a previous study [20]. The logistic regression analysis was therefore repeated using different cutoffs for ABI. For most of the cutoff values (<0.9, ≤0.91, ≤0.92, ≤0.93, ≤0.94 or ≤0.95), ABI remained an independent predictor of stroke (S1 Table). However, ABI ≤0.95 was observed in only 2.96% of the study participants, and the prevalence of stroke in those with an ABI ≤0.95 was only 25.0% (11 of 44), compared with a value of 34.6% (9 of 26) in those with an ABI <0.9 (*p* < 0.01). This suggests that ABI <0.9 correlates better with the prevalence of stroke than these other cutoff values.

ROC curve analysis was performed as an additional means of determining the optimal ABI cutoff for prediction of stroke. Comparison of the area under the ROC curve (ROC_AUC_) for various ABI cutoff values (0.9, 0.91, 0.92, 0.93, 0.94 and 0.95) revealed no significant differences between ABI = 0.90 and ABI = 0.91, 0.94 and 0.95 (S2 Table). Furthermore, ROC_AUC_ was significantly smaller for ABI = 0.92 and 0.93 than for ABI = 0.90. These findings indicate that the optimal cutoff value for ABI was 0.9, even though the use of this cutoff appeared to have low sensitivity and specificity for the prediction of stroke prevalence.

Given the apparent limitations of ABI in predicting stroke prevalence, we assessed discrimination and reclassification to evaluate the contribution of an interankle BP difference ≥10 mmHg to the prediction of stroke risk. First, the incremental value of the interankle BP difference was tested by comparing ROC_AUC_ values. ROC_AUC_ was estimated for a regression model with independent risk factors for stroke (age, family history of stroke, hypertension and ABI), and for a model consisting of the same factors plus interankle BP difference as a categorized variable, both in relation to a dichotomous outcome of history of stroke (with versus without). The ROC_AUC_ was 0.78 (95% CI = 0.75–0.81) for the model incorporating the independent risk factors alone, and 0.79 (95% CI = 0.75–0.82) when both independent risk factors and interankle BP difference were included (*p* = 0.0784) (S1 Fig).

NRI analyses were also undertaken to further establish whether the addition of interankle BP difference to the factors (including ABI) independently associated with stroke (identified in the logistic regression analysis) would improve the prediction of stroke prevalence. The results of this analysis are presented in Table 3. When interankle BP difference (≥10 mmHg vs. <10 mmHg) was used in addition to the other variables, 25.74% of participants with stroke were correctly reclassified to a higher risk category, and 5.88% were incorrectly reclassified to a lower risk category. Similarly, when interankle BP difference was used, 2.22% of participants without stroke were correctly moved down to a lower risk category, and 10.16% were incorrectly moved up to a higher risk category. These reclassification rates gave an estimated NRI of 11.92%. Thus, the addition of interankle BP difference ≥10 mmHg may improve the prediction of stroke by the combination of independent risk factors (age, family history of stroke, hypertension, and ABI).

**Table 3:** Net reclassification improvement by adding interankle blood pressure difference to the risk factors independently associated with stroke (age, family history of stroke, hypertension, and ankle-brachial blood pressure index).

	Reclassified predicted risk (with IAΔBP)				Reclassified (%)		
Predicted risk(without IAΔBP)	<10%	10 to<20%	20 to<30%	≥30%	Increased risk	Decreased risk	Net correctly Reclassified
**With stroke (*n* = 136)**							
**<10%**	**34**	**8**	**0**	**0**	**25.74%**	**5.88%**	**19.86%**
**10 to <20%**	**0**	**43**	**27**	**0**	**(35)**	**(8)**	
**20 to <30%**	**0**	**0**	**1**	**0**			
**≥30%**	**0**	**0**	**8**	**15**			
**Without stroke (*n* = 1349)**							
**<10%**	**962**	**58**	**0**	**0**	**10.16%**	**2.22%**	**7.94%**
**10 to 20%**	**0**	**188**	**79**	**0**	**(137)**	**(30)**	
**20 to 30%**	**0**	**0**	**0**	**0**			
**≥30%**	**0**	**0**	**30**	**32**			
**NRI (95% CI)**		**11.92 (2.89–20.95), *p* = 0.00966**					

Abbreviations: CI, confidence interval; IAΔBP, interankle blood pressure difference.

## Discussion

In the present study, we evaluated the association between prior stroke and BP ratio and differences obtained from simultaneous four-limb BP measurement. The major finding was that an ABI <0.9, but not an interarm BP difference ≥15 mmHg or an interankle BP difference ≥10 mmHg, is an independent risk factor for stroke prevalence in Chinese adults. However, the addition of an interankle BP difference ≥10 mmHg to the independent risk factors for stroke (age, family history of stroke, hypertension and ABI) improved the prediction of stroke (a net 11.92% reclassification improvement).

Our study is the first Chinese population-based cross-sectional observational study of the association of four-limb BP with stroke. We used the widely accepted ABI cutoff of <0.90 from clinical and epidemiologic studies as a criterion for a low ABI, and further established (by repeat logistic regression analyses and ROC curve analysis) that this cutoff was superior to alternative values ranging from 0.91 to 0.96. The prevalence of ABI <0.9 was 1.82% in Chinese adults ≥35 years old, which is lower than the 5% previously reported in a general population [21]. In the present study, 34.62% of participants with ABI <0.9 had a history of stroke. Participants with ABI <0.9 had a significantly higher prevalence of stroke than those with ABI >0.9 (34.62% vs. 8.79%). Using multivariable analysis adjusted for other traditional confounding factors, we confirmed that ABI <0.9 was an independent risk factor for the prevalence of stroke in Chinese adults. Our finding is in line with the results of a large national screening database [22]. A previous report from the Atherosclerosis Risk In Communities (ARIC) study demonstrated that ABI was inversely associated with stroke prevalence in primary care cohorts [23], which were subject to selection bias, compared to our simple random sampling population study according to age group and sex. Intracranial atherosclerosis is known to be a common cause of stroke. Low ABI has been reported to be associated with factors related to generalized atherosclerosis, including common carotid artery intima–media thickness, and stenosis in the intracranial internal carotid artery and middle cerebral artery [24, 25]. These studies provide pathologic evidence in support of our present study. The results of our logistic regression analysis indicate that some traditional stroke risk factors, including age, family history of stroke and hypertension, remained significant for prevalent stroke even after measurement of low ABI.

However, ABI has its limitations. The prevalence of ABI <0.9 in the study participants (1.75%) was much lower than that of a history of stroke (9.16%), indicating that ABI <0.9 may not be a sensitive predictor of stroke, as reported previously [20]. Also, ABI varies with gender and ethnicity, and so a single cutoff value may not be appropriate for all individuals [26]. Furthermore, ABI cannot be correctly evaluated in some patients, such as those with kidney failure who have an artificial arteriovenous fistula in one arm. In patients with stenosis in the ipsilateral brachial artery and ankle artery, ABI may be “pseudonormal.” In these cases, it is interesting to observe whether interarm or interankle BP difference can provide valuable relevant information concerning stroke risk.

Our study showed that, though participants with an interarm BP difference ≥15 mmHg had a higher prevalence of stroke than those with values <15 mmHg (21.21% vs. 8.97%), the difference was not independently associated with a history of stroke. The prevalence of interarm BP difference ≥10 mmHg was 11.46%, and that for ≥15 mmHg was 3.03%. This is consistent with a large community-based cohort of middle-aged men and women, without cardiovascular disease, in which an interarm systolic BP difference ≥10 mmHg was present in nearly 10%, and a difference ≥15 mmHg was present in 2.1% [27]. We used an interarm BP difference ≥15 mmHg as the cutoff value. Our study is the largest thus far to use a simultaneous measurement method, and we found that an interarm difference of ≥15 mmHg was not an independent risk factor for stroke. It would be useful to investigate the relationship between interarm BP difference and stroke in the same population, to use both simultaneous and non-simultaneous measurement. In a hospital-based retrospective observational study of interarm BP difference in patients with acute ischemic stroke, where atherosclerosis was determined by cerebral angiography covering both intracranial and extracranial cerebral arteries, the presence of a systolic BP difference ≥10 mmHg was not associated with atherosclerosis in the cerebral artery [28].

Unfortunately, there is no universally accepted diagnostic criterion for interankle BP difference [13, 29], and there are few data concerning the relationship between interankle BP difference and stroke. In our study, the prevalence of increased interankle systolic BP difference was found to be 31.09% for ≥10 mmHg and 17.19% for ≥15 mmHg. Participants with an interankle BP difference ≥10 mmHg had a higher prevalence of stroke than those with a difference <10 mmHg (17.73% vs. 12.58%). It is an interesting finding that interankle BP difference ≥10 mmHg may be a risk factor associated with stroke after adjustment for traditional risk factors and interarm BP difference ≥15 mmHg. It is suggested that ABI <0.9 and high BP may influence the association between interankle BP difference and stroke. Another notable observation of the present study is that the addition of an interankle BP difference ≥10 mmHg to the independent risk factors for stroke (age, family history of stroke, hypertension, and ABI) improved the prediction of stroke. An interankle BP difference ≥10 mmHg tends to reclassify appropriately participants with stroke from lower to higher risk state. This is suggested that current risk assessment tools underestimate the prevalence of stroke. However, the above finding is necessary to be confirmed. The precise mechanism causing the differences in vascular function remains unclear. From the above results, we speculate that atherosclerosis in the lower limb rather than upper limb arteries may be more relevant to cranial artery atherosclerosis.

In addition, there are data suggesting that individuals with an abnormal simultaneous BP measurement have a higher prevalence of stroke than those with normal measurements: 77.94% of participants with a history of stroke had at least one abnormal simultaneous four-limb BP measurement, including brachial BP, ABI, interarm BP difference and interankle BP difference. This finding may be important for improving primary stroke prevention strategies.

A few limitations of our study need to be mentioned. First, the BP ratio and differences in this study focused on systolic BP, rather than diastolic BP. One of the reasons is that decreased diastolic BP in isolated systolic hypertension may underestimate interarm and interankle BP differences. Second, the criterion of ≥10 mmHg used to define elevated interankle BP difference was somewhat arbitrary. Future studies are planned in this population that will include a comprehensive evaluation of the optimal cutoff value for interankle BP difference. Third, the number of participants with stroke was still relatively small, which prevented us from performing subgroup analyzes for ischemic stroke and hemorrhage stroke. Fourth, since we could not determine whether the participants’ plasma glucose level was ≥11.1 mmol/L at any time, the prevalence of diabetes mellitus could have been underestimated. Hence, diabetes mellitus was excluded from the traditional risk factors for stroke for the purposes of multivariable logistic regression analysis. Fifth, as this was a cross-sectional study carried out at one time point, it gives no indication of the sequence of events, and hence it is impossible to infer causality between the measured parameters (risk factors) and stroke. Moreover, it is possible that survival selection bias may have influenced our observations and conclusions. It is our intention to follow up with the participants of this Chinese cohort and explore whether ABI, or ABI combined with inter-ankle BP difference, can predict the incidence of stroke, and determine the appropriate predictive cutoff value for ABI. Nonetheless, large-scale prospective studies are merited to confirm and extend our findings.

In conclusion, simultaneous four-limb BP measurements may help physicians to identify rapidly the individuals at risk of stroke in a Chinese population. ABI <0.9, rather than interarm BP difference ≥15 mmHg or interankle BP difference ≥10 mmHg, is an independent risk factor for prevalent stroke in Chinese adults. The addition of interankle BP difference ≥10 mmHg to the independent risk factors for stroke may improve the prediction of stroke. It is necessary to establish a cohort study to investigate simultaneous four-limb BP measurements and stroke. Imaging studies are required to investigate the arterial structural characteristics of patients with abnormal interarm or interankle BP differences.

## Supporting Information

S1 Data The primary data of the study population. (RAR)

S1 Fig The incremental prognostic value of interankle BP difference ≥10 mmHg. (TIFF)

S1 Table Multivariate logistic regression analysis of the association between ankle-brachial blood pressure index (ABI) at various cutoffs and the prevalence of stroke. ABI, ankle-brachial blood pressure index; CI, confidence interval.
(DOCX)

S2 Table Comparison of ROC_AUC_ values for various ABI cutoffs. (DOCX)
