# Supplementary figures and images for: The Association of Four-Limb Blood Pressure with History of Stroke in Chinese Adults: A Cross-Sectional Study
Source: PLoS One. 2015 Oct 9;10(10):e0139925. doi: 10.1371/journal.pone.0139925 (PMC4599855; doi:10.1371/journal.pone.0139925)

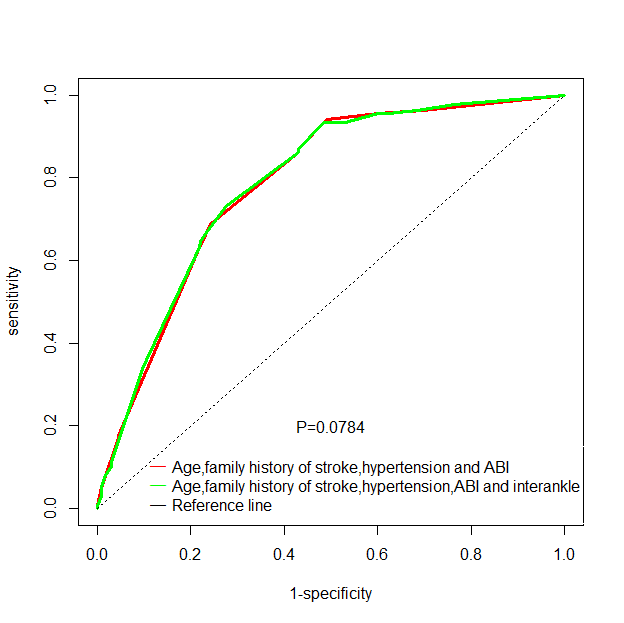

Supplement: S1 Fig — (TIFF) [file pone.0139925.s002.tiff]
