# Supplementary material for: The Association of Four-Limb Blood Pressure with History of Stroke in Chinese Adults: A Cross-Sectional Study
Source: PLoS One. 2015 Oct 9;10(10):e0139925. doi: 10.1371/journal.pone.0139925 (PMC4599855; doi:10.1371/journal.pone.0139925)
Supplement: S1 Table — ABI, ankle-brachial blood pressure index; CI, confidence interval. (DOCX) [file pone.0139925.s003.docx]

**S1 Table.** Multivariate logistic regression analysis of the association between ankle-brachial blood pressure index (ABI) at various cutoffs and the prevalence of stroke. ABI, ankle-brachial blood pressure index; CI, confidence interval.

| ABI cutoff | Odds ratio | 95% CI | *p* |
| --- | --- | --- | --- |
| <0.90 | 0.835 | 0.748～0.932 | 0.0014 |
| ≤0.91 | 0.842 | 0.756–0.938 | 0.0019 |
| ≤0.92 | 0.885 | 0.800–0.977 | 0.0161 |
| ≤0.93 | 0.895 | 0.811–0.988 | 0.0272 |
| ≤0.94 | 0.887 | 0.807–0.974 | 0.0125 |
| ≤0.95 | 0.907 | 0.833–0.988 | 0.0248 |
| ≤0.96 | 0.923 | 0.850­1.002 | 0.0573 |
