# Supplementary material for: The Association of Four-Limb Blood Pressure with History of Stroke in Chinese Adults: A Cross-Sectional Study
Source: PLoS One. 2015 Oct 9;10(10):e0139925. doi: 10.1371/journal.pone.0139925 (PMC4599855; doi:10.1371/journal.pone.0139925)
Supplement: S2 Table — (DOCX) [file pone.0139925.s004.docx]

**S2 Table.** Comparison of ROC_AUC_ values for various ABI cutoffs.

| ABI | ROC_AUC_ | *p* |
| --- | --- | --- |
| 0.90 | 0.5268 |  |
| 0.91 | 0.5264 | 0.3173 |
| 0.92 | 0.5246 | 0.01413 |
| 0.93 | 0.5242 | 0.00801 |
| 0.94 | 0.5271 | 0.9293 |
| 0.95 | 0.5282 | 0.7917 |

ABI, ankle-brachial blood pressure index; ROC_AUC_, area under the receiver operating characteristics curve. The *p* values are for comparisons of ROC_AUC_ with that for an ABI cutoff of 0.9.
